# Biomarkers and Mechanism Analysis for Polygoni Multiflori Radix Preparata-Induced Liver Injury by UHPLC-Q-TOF-MS-Based Metabolomics

**DOI:** 10.1155/2021/7677392

**Published:** 2021-11-23

**Authors:** Liming Wang, Zhida Wang, Yanchao Xing, Erwei Liu, Xiumei Gao, Linlin Wang, Zhifei Fu

**Affiliations:** ^1^State Key Laboratory of Component-based Chinese Medicine, Tianjin University of Traditional Chinese Medicine, 10 Poyanghu Road, Jinghai, Tianjin 301617, China; ^2^NHC Key Laboratory of Hormones and Development, Tianjin Key Laboratory of Metabolic Diseases, Chu Hsien-I Memorial Hospital & Tianjin Institute of Endocrinology, Tianjin Medical University, Tianjin 300070, China; ^3^Second Affiliated Hospital of Tianjin University of Traditional Chinese Medicine, Tianjin 300250, China

## Abstract

**Background:**

Polygonum Multiflorum Radix Preparata (PMP), prepared from *Polygonum multiflorum* Thunb. (PM), is traditionally valued for its liver and kidney-tonifying effects. However, the previous studies showed that PMP was hepatotoxic, which limited its clinical use. Unfortunately, the potential hepatotoxic ingredients and the molecular mechanism are still uncertain.

**Objective:**

The aim of this study was to find out potential biomarkers of hepatotoxicity using metabolomics profile.

**Materials and Methods:**

60% ethanol extract of PMP (PMPE) was prepared. Subsequently, an untargeted metabolomics technology in combination with ROC curve analysis method was applied to investigate the alteration of plasma metabolites in rats after oral administration of PMPE (40 g/kg/d) for 28 days.

**Results:**

Compared to the control group, the significant difference in metabolic profiling was observed in the PMPE-induced liver injury group, and sixteen highly specific biomarkers were identified. These metabolites were mainly enriched into bile acids, lipids, and energy metabolisms, indicating that PMPE-induced liver injury could be related to cholestasis and dysregulated lipid metabolism.

**Conclusions:**

This study is contributed to understand the potential pathogenesis of PMP-induced liver injury. The metabonomic method may be a valuable tool for the clinical diagnosis of PMP-induced liver injury.

## 1. Introduction

Polygoni Multiflori (PM, Heshouwu in Chinese), derived from the roots of *Polygonum multiflorum* Thunb., is a widely used Chinese medicinal material in clinic. Raw PM (RPM) has the effects of detoxicating, eliminating carbuncle, cutting off malaria, loosening bowel, and relieving constipation. A few of ancient books of traditional Chinese medicine, such as “ben cao hui yan,” recorded that RPM is poisonous [1]. To reduce toxicity and enhance tonic effect, RPM was usually processed by black bean sauce or by nine cycles of steaming and sun drying to obtain the processed PM (PMP) [2]. PMP demonstrates the functions of nourishing the liver, kidney, and blood, strengthening the muscles and bones, and blackens hair [3–6]. Interestingly, RMP steamed with black soybeans for nine cycles was safe. However, modern processing technologies, usually steamed with the black soybean juice once, still exhibit obvious hepatotoxicity [7, 8]. PMP-related liver injury has been reported in many countries and regions, such as South Korea, Japan, and UK [9]. Recently, the National Medical Products Administration in China has also warned of the risk of liver injury from the extracts of RPM and PMP [10]. Unfortunately, although extensive experiments have been performed in recent years, the potential toxic mechanisms that cause liver injury remain unclear. Therefore, it is particularly urgent and important to establish a method for the early warning of liver injury of PMP.

Metabolome is located downstream of gene regulation and protein network and could provide terminal information of biology. Metabolomics is the study of endogenous metabolite alterations associated with maintaining the normal function and development of organisms after the stimulation or disturbance of biological systems [11]. It has been widely applied in various fields of life sciences and represents a powerful tool for interpreting life phenomena and exploring mechanisms of disease [12]. Chromatography combined with mass spectrometry (such as LC-MS and GC-MS) and nuclear magnetic resonance (NMR) spectroscopy is widely applied in metabolomics studies, and LC-MS is the most popular used methodology in untargeted metabolomics research. Ultrahigh performance liquid chromatography-mass spectrometry technology provides a rapid, concise, and high-throughput approach for the analysis of biological fluids or tissues [13]. Combined with multivariate statistical analysis methods, it provides an effective method for the discovery of metabolic pathways. The targeted and untargeted methods in MS-based metabolomics were used to detect changes in the processing technologies of Polygoni Multiflori Radix [14]. In conclusion, metabolomics has been extensively used for the discovery of biomarkers and the research on the metabolic mechanism [15].

The hepatotoxicity components of PMP have always been the subject of controversy. In our previous study [16], we performed a comparative study on the chemical components in the aqueous extract and 60% ethanol extract of PMP (PMPE). The results showed that the different components were attributed to anthraquinones, such as emodin, rhein, and physcion. Furthermore, the pharmacokinetics characters of these main components in rats were studied. The *t*_1/2(h)_ of those components of the PMPE was longer compared to the aqueous extract of PMP and increased with the increasing of dosage of PMPE. Anthraquinone components (such as emodin) which has higher content in PMPE are regarded as potential liver damage components [16]. Based on our previous research and literature review, the PMPE showed a degree of hepatotoxicity [4, 16, 17]; therefore, we selected PMPE to investigate the components and mechanism of potential liver injury of PMP. The study aimed to further explore the potential hepatotoxicity mechanism of PMPE using the LC-MS-based metabolomics method. The reliability of discriminated metabolite was further screened by receiver operating characteristic (ROC) curve analysis, and the related metabolic pathways were clarified. Combined with biochemical indicators, our work could further clarify the potential mechanism of PMPE-induced liver injury.

## 2. Materials and Methods

### 2.1. Chemicals and Reagents

HPLC grade methanol (ME) and acetonitrile (ACN) were provided by Thermo Fisher Scientific Co., Ltd. (Pittsburgh, PA, USA). Ultrapure water was obtained from Watsons Food&Beverage Co., Ltd. (Guangdong, China). PMP was purchased from Anguo Shengshan Pharmaceutical Co., Ltd. (Hebei, China).

### 2.2. Sample Preparation

PMP (10 kg) was extracted with 60% ethanol (v/v) to yield 1.7 kg extract (PMPE), stored at 4°C for further use. The main constituents in PMPE were profiled and reported by our previous research [16].

### 2.3. Animal and Experiment Design

Sprague Dawley (SD) rats (6–8 weeks) were purchased from HFK Bioscience Co., Ltd. (Beijing, China). All animals were kept under the same breeding room conditions with a temperature between 20 and 25°C and given standard laboratory water and food. The procedures involving animals and their care were conformed to the Guiding Principles for the Care and Use of Laboratory Animals of China. The rats were kept in the breeding room for 7 days and then randomly divided into different groups (*n* = 7 for each group).

Animals were randomly divided into the blank control group (BC, equivalent physiological saline) and PMPE treatment group; based on our previous studies [16], the dosage was set at 40 g/kg per day (amount of crude drug weight of rat/day). The clinical syndromes of animals were observed every day. The animal's body weight was monitored once a week after intragastric administration.

Rats were fasted for 12 hours after oral administration of PMPE for 28 days, and blood sample of rat was collected from the abdominal aorta. One part with no anticoagulant samples remained for 2 hours, 3500 × g was centrifuged for 10 min, and then serum was obtained for analysis of the serum biochemical index. The remaining serum with anticoagulant was centrifuged under 3000 × g for 10 min, and finally, the plasma was obtained and frozen in the refrigerator at –80°C for further analysis. The liver of each rat was taken for histological examination, and the organ index was calculated.

### 2.4. Serum Biochemical Analysis

The serum biochemical indexes, including alanine aminotransferase (ALT), aspartate aminotransferase (AST), total bile acid (TBA), alkaline phosphatase (ALP), total bilirubin (T-BIL), and triglyceride (TG), were measured dynamically using a Hitachi 7020 automatic biochemistry analyzer (Tokyo, Japan).

### 2.5. Liver Histopathology Assessment

The liver samples were fixed with 10% neutral formalin for 48–72 h, embedded in paraffin after fixation, continuously sectioned at a thickness of 5 *µ*m, stained with hematoxylin and eosin (H&E), and evaluated using a microscope.

### 2.6. Plasma Preparation and UHPLC/Q-TOF-MS Analysis

An amount of 100 *μ*L of plasma was added into 400 *μ*L of acetonitrile to precipitate protein. All the samples were vortexed for 5 min and centrifuged at 14000 × g for 10 min under 4°C. The supernatant was transferred into 1.5 mL tube and dried under a nitrogen stream. All samples were redissolved in 100 *μ*L of methanol-water (80 : 20, v/v). Each reconstituted sample was centrifuged at 14000 × g for 10 min under 4°C, and then the supernatant was transferred into sample vials for analysis using UHPLC/Q-TOF-MS. The quality control (QC) samples were prepared by adding equal amounts of all analytes. During the research process, QC was carried out every five samples to verify the stability and accuracy of the system.

LC analysis was conducted on an Agilent 1290 UHPLC system equipped with an ACQUITY UPLC HSS T_3_ column (2.1 × 100 mm, 1.8 *µ*m, Waters, Ireland) under the temperature of 35°C. The mobile phase consisted of 0.1% formic acid in water (A) and acetonitrile (B). The gradient of elution conditions was as follows: 0–13 min, 3–100% B, 13–17 min, 100% B. The flow rate was 0.4 mL·min^−1^, and the injection volume was 2 *μ*L.

The mass spectrometry analysis was performed on an Agilent 6520 Accurate-Mass Q-TOF/MS system. The conditions of the ESI source were drying gas flow rate, 8.0 L·min^−1^; drying gas temperature, 350°C; nebulizer, 30 psi; capillary voltage (Vcap), 4000 V in positive ion mode, and 3500 V in negative ion mode; fragmentor voltage, 150 V and 175 V in positive and negative ion modes, respectively. Collision energy was 20 V and 40 V, and the scanning range of the mass analyzer was *m/z* 50–1000.

### 2.7. MS Data Processing Analysis

Data collection and procession were performed using the following software and databases: Agilent Mass Hunter Workstation software (version B.04.00) and Agilent MSC software (version B.07.00). Metabolites were identified through precise molecular weight, retention time, MS/MS fragmentation, the Metlin database (http://metlin.scripps.edu/index.php), and HMDB database (https://hmdb.ca/). The precursor ion mass tolerance was set to 10 ppm.

All data were normalized using the sum method. Subsequently, multivariate data analysis was conducted using the SIMCA-P (V 14.1, Umetrics, Sweden), including principal component analysis (PCA), partial least squares discriminant analysis (PLS-DA), and orthogonal partial least squares discriminant analysis (OPLS-DA). Permutation test was used to evaluate whether the established model was overfitting. A cross-validated analysis of variance was used to evaluate the reliability of the OPLS-DA model.

### 2.8. Statistical Analysis

All data were expressed as mean ± standard deviation, and the data were analyzed using GraphPad Prism version 8.01 (GraphPad Software Inc., La Jolla, CA, USA). Student's *t*-test was used to assign significance (*p* < 0.05).

## 3. Results

### 3.1. Clinical Observation after Treatment of PMPE

**Figure 1:**
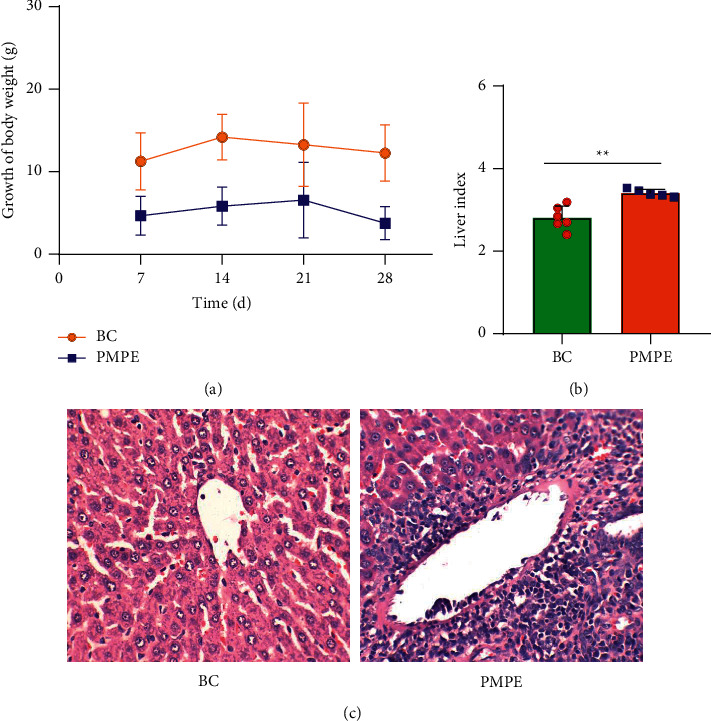
The weight curve within 28 days of rats (a), liver index (b), and (c) Pathological sections of liver of rats after 28 days of administration of 60% ethanol extract of PMP (400 times under light microscope) in Blank Control (BC) and 60% ethanol extract of PMP (PMPE) groups ^*∗*^*p* < 0.05, compared with the control group, ^*∗∗*^*p* < 0.01, compared with the control group.

Compared with the BC group, the weekly growth of the body weight in PMPE-treated rats significantly reduced (Figure 1(a)), whereas the liver index of PMPE-treated rats significantly increased (*p* < 0.05) (Figure 1(b)). Liver pathology revealed liver cell degeneration, necrosis, and inflammatory cell infiltration after administration PMPE (Figure 1(c)).

### 3.2. Hepatotoxicity Assessment of the PMPE

**Figure 2:**
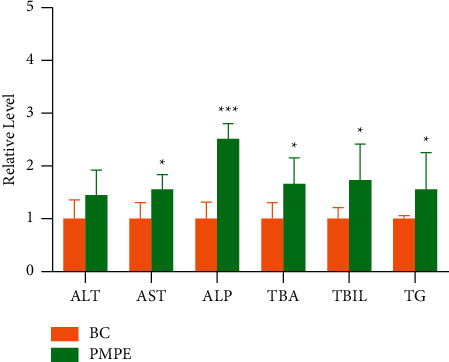
Serum relative levels of ALT, AST, ALP, TG, TBA, and TBIL in BC and PMPE groups, ^*∗*^*p* < 0.05, ^*∗∗∗*^*p* < 0.0001 compared with the control group. ALT, alanine aminotransferase; AST, aspartate aminotransferase; ALP, alkaline phosphatase; TG, triglyceride; TBA, total bile acid; TBIL total bilirubin.

Compared with the BC group, the levels of ALP, TAB, and TBIL increased significantly (*p* < 0.05), and the content of ALT, TG, and AST also showed increasing tendency (Figure 2).

### 3.3. Metabolomic Profile Analysis of PMPE-Induced Liver Injury

A total of 1827 and 992 features in plasma were extracted in the positive and negative ion mode, respectively. A representative plasma total ion current (TIC) chromatogram of the blank control group and the PMPE group are shown in Supplementary Figure S1.

### 3.4. Precision and System Stability Analysis

In order to estimate the precision and stability of the analytical method, QC samples were included into the entire analysis process, and the peak areas of the 6 characteristic ions were selected as evaluation indicators. As shown in Supplementary Table S1, the relative standard deviation (RSD) values of the peak area of the six characteristic ions of the QC samples were all less than 10%.

### 3.5. Identification of Potential Liver Injury Biomarker and Pathway Analysis

**Figure 3:**
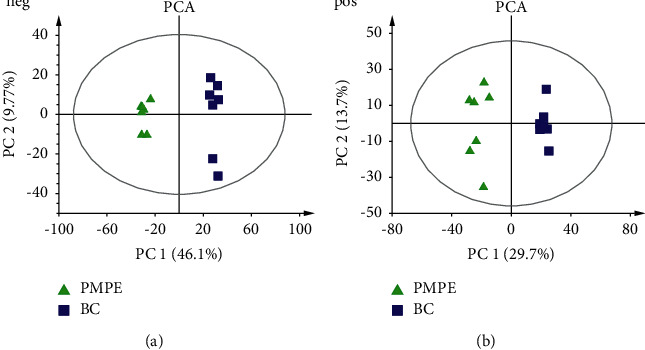
PCA score plots of the LC-MS spectra from plasma. Negative (left) and Positive (right).

**Figure 4:**
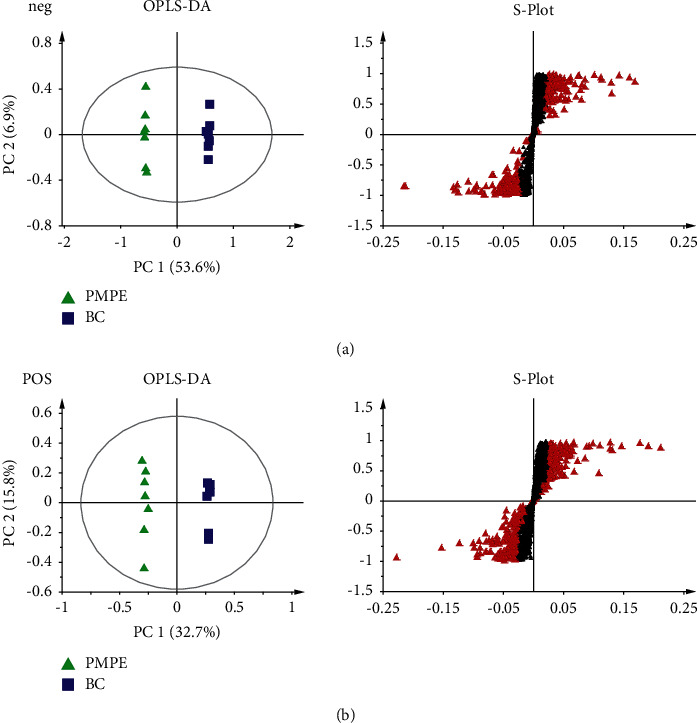
OPLS-DA analysis (left) and S-Plot (right) of the rat plasma from LC−MS spectra negative mode and positive mode.

PCA was used for visualizing group clustering and visualizing possible outliers. The BC group and PMPE group were obviously separated in the positive and negative ion mode (positive: *R*^2^ = 0.433, *Q*^2^ = 0.177; negative: *R*^2^ = 0.559, *Q*^2^ = 0.344), indicating that there was a significant difference between the BC and the PMPE groups (Figure 3). PLS-DA and permutation analysis were performed to evaluate the reliability of the model (positive: *R*^2^ = 0.487, *Q*^2^ = 0.96; negative: *R*^2^ = 0.605, *Q*^2^ = 0.99). The model validation of PLS-DA was performed at 200 permutations analysis (Supplementary Figure S1), and the results indicated that the PLS-DA model was credible and had no overfitting. OPLS-DA was carried out to reveal the differences of metabolites among groups as a supervised classification analysis method (positive: *R*^2^ = 0.568, *Q*^2^ = 0.952; negative: *R*^2^ = 0 .605, *Q*^2^ = 0.987). Scatter plots and S-plot are shown in Figure 4. Variable important in projection (VIP) was used to evaluate the strength and explanatory power of the expression pattern of metabolites on the classification and discrimination of each group of samples. Differential metabolites were screened according to the VIP (VIP > 1.0) and *P* values of Student's *t*-test (*p* < 0.05). A total of 342 differential characteristic ions from positive and negative ion patterns were selected. Based on the exact molecular ion mass, retention time of reference compounds, and metabolites database (METLIN, LIPID MAPS, and HMDB database), a total of 39 metabolites were identified as biomarker candidates of PMPE-induced liver injury finally. All candidate biomarkers were presented in Supplementary Table S2.

**Figure 5:**
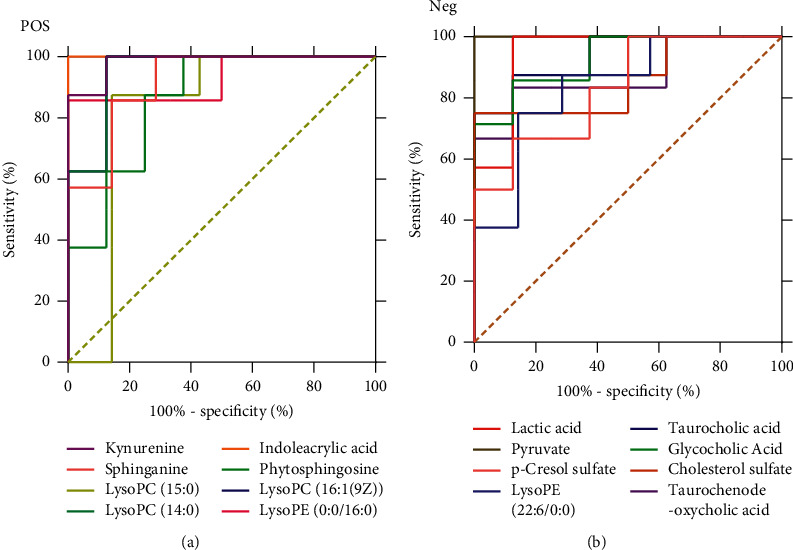
Potential biomarker ROC curve analysis. Positive (left) and Negative (right).

**Figure 6:**
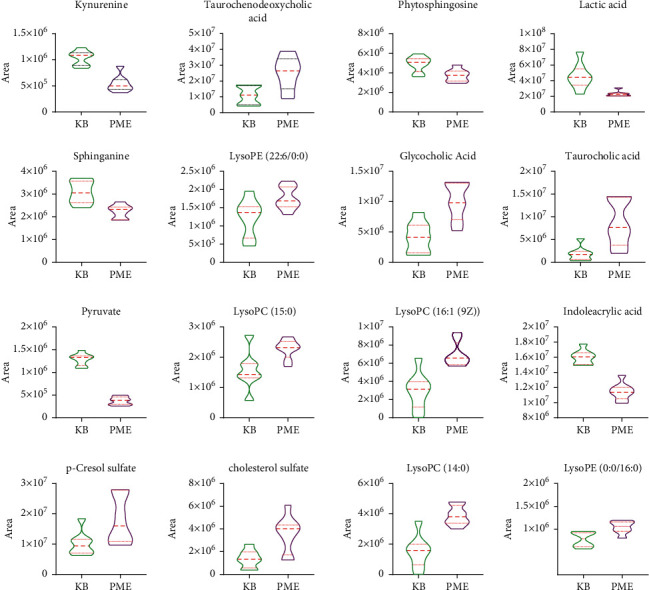
The relative level of potential biomarker content.

The ROC curves were performed for the analysis of sorting out the potential diagnostic metabolites with high specificity and sensitivity to distinguish between the BC and PMPE groups. The diagnostic values were assessed by the area under the ROC curve (AUC), which was carried out using GraphPad Prism (version 8.0 San Diego, USA). The AUCs of candidate liver injury biomarkers which were higher than 0.8 were regarded as potential biomarkers (Figure 5 and Supplementary Table S3). The results manifested that these biomarkers had better discrimination ability for healthy rats and liver injury rats. A total of 16 potential biomarkers were reconfirmed from the positive and negative ion patterns in rat plasma (8 for negative and 8 for positive). Potential biomarkers are summarized in Table 1. Six metabolites were significantly decreased in the PMPE group compared with those in the BC group, including kynurenine, lactic acid, pyruvate, sphinganine, phytosphingosine, and indoleacrylic acid (Figure 6).

**Figure 7:**
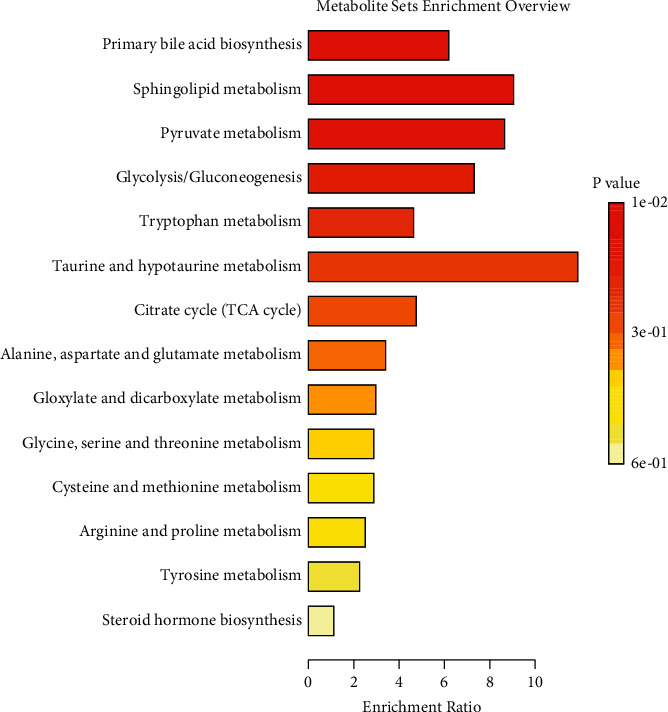
Differential metabolites pathway enrichment analysis of PMPE-induced liver injured.

**Figure 8:**
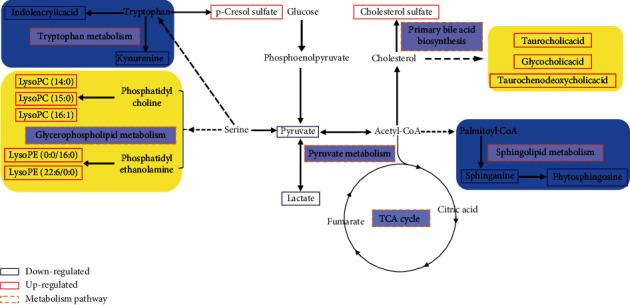
Potential metabolic pathway of PME-induced liver injured.

To further elucidate the metabolic pathways of specific metabolites associated with PMPE-induced liver injury, we used the MetaboAnalyst4.0 (https://www.metaboanalyst.ca/) for enrichment and topological analysis of the KEGG signaling pathway. The most affected metabolic pathways were primary bile acid biosynthesis, sphingolipid metabolism, energy metabolism, glycerophospholipid metabolism, and tryptophan metabolism (Figure 7 and Supplementary Figure S2). In order to exhibit the results clearly, we constructed a network of potential metabolic pathways based on biomarkers in rat plasma (Figure 8).

## 4. Discussion

Biliary cholestasis and bile duct injury are the main clinical manifestations of cholestasis caused by drugs. Serum biochemical parameters ALP is preferred in clinical diagnosis. According to the above results, the levels of ALP, TBA, and TBIL showed significantly increased accompanied by liver cell degeneration, necrosis, and inflammatory cell infiltration, indicating that the PMPE-induced hepatotoxicity may be related to cholestasis. Meanwhile, the levels of taurochenodeoxycholic acid, taurocholic acid, and glycocholic acid were significantly increased in plasma of hepatotoxicity rats. Pathway enrichment and topology analysis also indicated that the biosynthesis pathway of bile acids was disrupted or interfered. In the process of generation, formation, transportation, and discharge of bile acid, any failure in those steps could cause cholestasis. Bile acid, especially hydrophobic toxic bile acids accumulation in liver cells, could induce inflammatory reactions by damaging mitochondria and lead to endoplasmic reticulum stress. These changes could directly destroy membranes of cell and organelle by virtue of detergent action and lead to hepatocyte injury finally. When hepatocytes are damaged, bile acid metabolism is impaired and reversely flows into blood [18, 19]. These explain the mechanisms of serum TBA increased in rat with PMPE-induced liver injury.

As the endogenous ligand of FXR (farnesoid X receptor), bile acid is not only an important substance in lipid digestion and absorption but also serves as a signal molecule to transmit information through FXR-mediated signaling pathways and participate in the regulation of lipid metabolism in the body. FXR could monitor the metabolism of triglycerides and fatty acids through a variety of mechanisms. Generally, when FXR was activated by bile acids, SREPB-1c (sterol regulatory element-binding protein-1c), which is a major transcription factor involved in fat synthesis genes, was downregulated by SHP (recombinant small heterodimer partner) signal pathway in order to inhibit lipid synthesis. The activity of LDLR (low-density lipoprotein receptor) and VLDLR (very low-density lipoprotein receptor) was upregulated to increase lipid oxidation consumption and reduced lipid accumulation in the liver thereby [20]. Thus, lipids will accumulate in the liver when the activity of FXR was downregulated in the condition of disordered intrahepatic bile acid metabolism.

Phosphatidylcholine (PC) and phosphatidylethanolamine (PE), two most affluent glycerophospholipids, play a pivotal biological function in regulating lipoprotein metabolism [21]. They can be metabolized by phospholipase A2 (PLA2) into LPC (lysophosphatidylcholine), LPE (lysophosphatidylethanolamine), and arachidonic acid, which plays a very important role in lipid inflammation [22]. Metabolic disorders of these lipids result in changes in membrane lipid composition and affect the physical properties and functional integrity of the membrane. These could lead to hepatocellular apoptosis, inflammation, and the progression of liver disease. In addition, studies have reported that LPC has a certain proinflammatory function and is a new type of inflammatory lipids [23]. The experimental studies *in vivo* have shown that LPC can induce hepatitis which is harmful to the liver. It can also induce hepatocyte lipid apoptosis by activating GPCRs (G protein-coupled receptors) and depolarization of mitochondrial membrane [24]. Many researches have shown that the incubation of primary hepatocytes with LPC could induce cell death by apoptosis [25]. In our study, LPC and LPE were the most increased lipids in the oral administration group of PME, and the accumulation of these metabolites caused hepatocyte dysfunction. Sphingolipids are an important ingredient of the biofilm structure. As the important active molecules in organisms, sphingolipids and their metabolites have been identified as new biomarkers of chronic hepatopathy disease and hepatocellular carcinoma (HCC) [26].

As the site of gluconeogenesis and glycogen synthesis, the liver plays an important role in regulating blood sugar levels. Bile acids could regulate glucose metabolism through FXR-SHP or bile acid-TGR5 (Takeda G protein-coupled receptor 5) signal pathway [27]. Some representative important metabolites related to energy metabolism and tryptophan metabolism were detected in this study, such as pyruvic acid, lactic acid, kynurenine, and indoleacrylic acid. These metabolites were significantly reduced. The low level of kynurenine is closely related to abnormal liver function and energy metabolism. It has been reported that drug-induced liver injury is related to the mitochondrial function and energy homeostasis [28]. Moreover, the decrease of indoleacrylic acid is related to the disorder of tryptophan metabolism in the liver. The parallels with our findings are striking. These results indicated that liver injury affected the disorder of these metabolic pathways. In conclusion, all results indicated that PMP affected metabolic disorder of bile acids, lipids, and glucose metabolism; impaired liver function; and could induce liver injury finally.

Compared with the control group, the content of p-cresol sulfate in the PME group was significantly increased. Cholesterol sulfate is a representative substance of uremic toxin produced by tyrosine. The results suggested that the kidney may be damaged in different degrees, which may be attributed to the aggravation of kidney metabolism after liver injury. In addition, the disorder of bilirubin metabolism and blood circulation could also lead to renal function decline (hepatorenal syndrome) [29].

## 5. Conclusion

In summary, LC-MS-based plasma metabolomics analysis combined with ROC curve analysis method provides an integrated view of the metabolic features of PMP-induced hepatoxicity. The differential metabolites were identified. Metabolic pathway analysis indicated that the bile acid biosynthesis pathway was the main pathways affected by PMP, which may be the cause of liver injury. The sphingolipid metabolism, energy metabolism, glycerophospholipid metabolism, and tryptophan metabolism pathway were also involved. These discriminating metabolites may help to understand the pathogenesis of liver injury and provide a good prospect for the clinical diagnosis of PMPE-induced liver injury.

## Figures and Tables

**Table 1:** Biomarker candidates of PMPE-induced liver injury.

	VIP	FC	m/z	Formula	Biomaker	HMDB	KEGG	*P*-value
Pos	4.98	0.72	188.06905	C_11_H_9_NO_2_	Indoleacrylic acid	HMDB00734	-	5.62E-05
	1.99	0.47	209.09043	C_10_H_12_N_2_O_3_	Kynurenine^*∗*^	HMDB0000684	C00328	2.84E-04
	1.41	0.79	302.30336	C_18_H_39_NO_2_	Sphinganine	HMDB0000269	C00836	4.11E-02
	1.62	0.76	318.29862	C_18_H_39_NO_3_	Phytosphingosine	HMDB0004610	C12144	3.47E-02
	1.62	1.82	454.29073	C_21_H_44_NO_7_P	LysoPE(0 : 0/16 : 0)	HMDB0011473	-	1.10E-02
	2.62	2.58	468.30649	C_22_H_47_NO_7_P	LysoPC(14 : 0)	HMDB0010379	C04230	2.33E-03
	1.07	1.65	482.32192	C_23_H_48_NO_7_P	LysoPC(15 : 0)	HMDB0010381	C04230	4.79E-03
	3.74	2.35	494.32224	C_24_H_49_NO_7_P	LysoPC(16 : 1(9Z))	HMDB0010383	C04230	4.65E-02

Neg	1.39	0.30	87.00888	C_3_H_4_O_3_	Pyruvate^*∗*^	HMDB0000243	C00022	3.10E-07
	5.7	0.47	89.02465	C_3_H_6_O_3_	Lactic acid	HMDB0000190	C00256	1.02E-02
	2.49	1.54	187.00644	C_7_H_8_O_4_S	p-Cresol sulfate	HMDB11635	C06677	4.23E-02
	2.05	2.38	464.30076	C_26_H_43_NO_6_	Glycocholic acid	HMDB0000138	C01921	1.22E-02
	1.14	2.30	465.30223	C_27_H_46_O_4_S	Cholesterol sulfate	HMDB0000653	C18043	1.42E-02
	2.33	1.80	496.27305	C_26_H_45_NO_7_S	Taurocholic acid	HMDB0000036	C05122	3.02E-02
	3.8	2.23	498.28706	C_26_H_45_NO_6_S	Taurochenodeoxycholic acid	HMDB0000951	C05465	3.30E-02
	1.05	1.50	524.27794	C_27_H_44_NO_7_P	LysoPE (22 : 6/0 : 0)	HMDB0011526	-	6.14E-03

Pos: positive; Neg: negative; FC: fold change; HMDB: human metabolome database; KEGG: Kyoto encyclopedia of genes and genomes. ^*∗*^: compared with the reference standards.

## Data Availability

Data are available from the corresponding author upon request.

## Abbreviations

PMP: Polygonum Multiflorum Radix Preparata
PM: Polygoni Multiflori
PMPE: 60% ethanol extract of PMP
ROC: Receiver operating characteristic
UHPLC-Q-TOF-MS: Ultrahigh performance liquid chromatography coupled with quadrupole time-of-flight mass spectrometry
QC: Quality control
RSD: Relative standard deviation.

## Supplementary Materials

Supplementary Materials Table S1: the relative standard deviation (RSD) values of the peak area of the six characteristic ions of the QC samples. Table S2: biomarker candidates of PMPE-induced liver injury in positive and negative mode. Table S3: the AUC values of candidate liver injury biomarkers. Figure S1: TIC of the LC-MS spectra, PLS-DA analysis, and permutations analysis in negative mode and positive mode. Figure S2: analysis of metabolic pathways related to liver injury.
