# Supplementary material for: Biomarkers and Mechanism Analysis for Polygoni Multiflori Radix Preparata-Induced Liver Injury by UHPLC-Q-TOF-MS-Based Metabolomics
Source: Evid Based Complement Alternat Med. 2021 Nov 23;2021:7677392. doi: 10.1155/2021/7677392 (PMC8632464; doi:10.1155/2021/7677392)
Supplement: Supplementary Materials — Table S1: the relative standard deviation (RSD) values of the peak area of the six characteristic ions of the QC samples. Table S2: biomarker candidates of PMPE-induced liver injury in positive and negative mode. Table S3: the AUC values of candidate liver injury biomarkers. Figure S1: TIC of the LC-MS spectra, PLS-DA analysis, and permutations analysis in negative mode and positive mode. Figure S2: analysis of metabolic pathways related to liver injury. [file 7677392.f1.docx]

Electronic Supporting Materials

**Biomarkers and Mechanisms Analysis for Polygoni Multiflori Radix Preparata-Induced Liver Injury by UHPLC-Q-TOF-MS** **Based Metabolomics**

# Liming Wang,^1#^ Zhida Wang,^2#^ Yanchao Xing,^1^ Erwei Liu,^1^

# Xiumei Gao,^1^ Linlin Wang,^3^ Zhifei Fu^1^

*^1^ State Key Laboratory of Component-based Chinese Medicine, Tianjin University of Traditional Chinese Medicine, 10 Poyanghu Road, Jinghai, Tianjin 301617, P. R. China*

*^2^ NHC Key Laboratory of Hormones and Development, Tianjin Key Laboratory of Metabolic Diseases, Chu Hsien-I Memorial Hospital & Tianjin Institute of Endocrinology, Tianjin Medical University, Tianjin, 300070, P. R. China*

*^3^ Second Affiliated hospital of Tianjin University of Traditional Chinese Medicine, Tianjin, 300250, P. R. China*

Correspondence should be addressed to Linlin Wang; lynnwlin@yeah.net and Zhifei Fu; fuzhifei@tjutcm.edu.cn

# Liming Wang and Zhida Wang contributed equally to this work.

**Table S1**

The relative standard deviation (RSD) values of the peak area of the six characteristic ions of the QC samples.

| **NO.** | **1** | **2** | **3** | **4** | **5** | **6** |
| --- | --- | --- | --- | --- | --- | --- |
| *m/z* | 240.06136 | 656.47498 | 303.23294 | 608.46533 | 239.05651 | 116.90592 |
| RT (s) | 156.31 | 704.11 | 714.8 | 714.66 | 156.38 | 143.25 |
| Peak Area RSD (%) | 8.79 | 8.02 | 5.56 | 9.58 | 7.53 | 6.89 |

**Table S2**

Biomarker candidates of PMPE-induced liver injury in positive and negative mode.

| Mode | NO | Compound | *m/z* | Formula | Adduct | VIP | P-value |
| --- | --- | --- | --- | --- | --- | --- | --- |
| POS | 1 | Vanillic acid | 169.04892 | C_8_H_9_O_4_ | [M+H]^+^ | 2.41 | 1.54E-05 |
|  | 2 | Indoleacrylic acid | 188.06905 | C_11_H_10_NO_2_ | [M+H]^+^ | 4.98 | 5.62E-05 |
|  | 3 | 3-Indolepropionic acid | 190.08472 | C_11_H_12_NO_2_ | [M+H]^+^ | 1.06 | 3.39E-03 |
|  | 4 | Tryptophan* | 205.09602 | C_11_H_13_N_2_O_2_ | [M+H]^+^ | 8.21 | 2.13E-05 |
|  | 5 | Kynurenine* | 209.09043 | C_10_H_13_N_2_O_3_ | [M+H]^+^ | 1.99 | 2.84E-04 |
|  | 6 | 5-phosphonooxy-L-lysine | 243.07424 | C_6_H_16_N_2_O_6_P | [M+H]^+^ | 1.01 | 2.22E-06 |
|  | 7 | Sphinganine | 302.30336 | C_18_H_40_NO_2_ | [M+H]^+^ | 1.41 | 4.11E-02 |
|  | 8 | Phytosphingosine | 318.29862 | C_18_H_40_NO_3_ | [M+H]^+^ | 1.62 | 3.47E-02 |
|  | 9 | Linoleyl carnitine | 424.33982 | C_25_H_47_NO_4_ | [M+H]^+^ | 1.41 | 3.38E-02 |
|  | 10 | LysoPE(0:0/16:0) | 454.29073 | C_21_H_45_NO_7_P | [M+H]^+^ | 1.62 | 1.10E-02 |
|  | 11 | LysoPC(14:0) | 468.30649 | C_22_H_48_NO_7_P | [M+H]^+^ | 2.62 | 2.33E-03 |
|  | 12 | LysoPC(15:0) | 482.32192 | C_23_H_49_NO_7_P | [M+H]^+^ | 1.07 | 4.79E-03 |
|  | 13 | LysoPE(0:0/18:0) | 482.32223 | C_23_H_49_NO_7_P | [M+H]^+^ | 1.07 | 4.65E-02 |
|  | 14 | LysoPC(16:1(9Z)) | 494.32224 | C_24_H_50_NO_7_P | [M+H]^+^ | 3.74 | 4.68E-03 |
|  | 15 | LysoPC(20:4) | 544.33829 | C_28_H_52_NO_7_P | [M+H]^+^ | 5.91 | 1.23E-03 |
|  | 16 | L-Urobilinogen | 597.36128 | C_33_H_49_N_4_O_6_ | [M+H]^+^ | 1.21 | 1.16E-02 |
| Mode | NO | Compound | *m/z* | Formula | Adduct | VIP | P-value |
| NEG | 1 | Pyruvate* | 87.00888 | C_3_H_3_O_3_ | [M-H]^-^ | 1.39 | 3.10E-07 |
|  | 2 | Lactic acid | 89.02465 | C_3_H_5_O_3_ | [M-H]^-^ | 5.70 | 1.02E-02 |
|  | 3 | β-hydroxy butyric acid | 103.03953 | C_4_H_7_O_3_ | [M-H]^-^ | 7.38 | 6.44E-05 |
|  | 4 | L-Phenylalanine* | 164.07024 | C_9_H_10_NO_2_ | [M-H]^-^ | 1.92 | 2.23E-03 |
|  | 5 | Homovanillic acid | 181.04923 | C_9_H_9_O_4_ | [M-H]^-^ | 1.37 | 3.98E-06 |
|  | 6 | p-Cresol sulfate | 187.00644 | C_7_H_7_O_4_S | [M-H]^-^ | 2.49 | 4.23E-02 |
|  | 7 | Phenylacetylglycine | 192.0651 | C_10_H_10_NO_3_ | [M-H]^-^ | 2.84 | 7.99E-03 |
|  | 8 | Lauric acid | 199.16896 | C_12_H_23_O_2_ | [M-H]^-^ | 1.63 | 3.62E-02 |
|  | 9 | Tryptophan | 203.08107 | C_11_H_11_N_2_O_2_ | [M-H]^-^ | 4.46 | 1.94E-08 |
|  | 10 | Palmitoleic acid | 253.2165 | C_16_H_29_O_2_ | [M-H]^-^ | 3.61 | 3.23E-03 |
|  | 11 | Palmitic acid | 255.23177 | C_16_H_31_O_2_ | [M-H]^-^ | 2.64 | 7.59E-05 |
|  | 12 | α-Linolenic Acid | 277.21651 | C_18_H_29_O_2_ | [M-H]^-^ | 3.32 | 1.80E-02 |
|  | 13 | Linoleic acid | 279.23274 | C_18_H_31_O_2_ | [M-H]^-^ | 5.89 | 1.52E-04 |
|  | 14 | Sphingosine-1-phosphate | 378.24108 | C_18_H_37_NO_5_P | [M-H]^-^ | 1.23 | 2.90E-04 |
|  | 15 | Chenodeoxycholic Acid | 391.28331 | C_24_H_39_O_4_ | [M-H]^-^ | 1.96 | 2.11E-04 |
|  | 16 | Chenodeoxyglycocholic acid | 448.3049 | C_26_H_42_NO_5_ | [M-H]^-^ | 1.35 | 1.60E-03 |
|  | 17 | LysoPE(0:0/16:0) | 452.27834 | C_21_H_43_NO_7_P | [M-H]^-^ | 2.59 | 5.96E-05 |
|  | 18 | Glycocholic Acid | 464.30076 | C_26_H_42_NO_6_ | [M-H]^-^ | 2.05 | 1.22E-02 |
|  | 19 | cholesterol sulfate | 465.30223 | C_27_H_45_O_4_S | [M-H]^-^ | 1.14 | 1.42E-02 |
|  | 20 | LysoPE(18:2/0:0) | 476.27835 | C_23_H_43_NO_7_P | [M-H]^-^ | 1.01 | 1.43E-02 |
|  | 21 | LysoPE(0:0/18:0) | 480.30923 | C_23_H_47_NO_7_P | [M-H]^-^ | 1.12 | 2.67E-07 |
|  | 22 | Taurocholic acid | 496.27305 | C_26_H_42_NO_6_S | [M-H_2_O]^-^ | 2.33 | 3.02E-02 |
|  | 23 | Taurochenodeoxycholic acid | 498.28706 | C_26_H_44_NO_6_S | [M-H]^-^ | 3.80 | 3.30E-02 |
|  | 24 | LysoPE(20:4/0:0) | 500.27845 | C_25_H_43_NO_7_P | [M-H]^-^ | 1.20 | 5.06E-02 |
|  | 25 | LysoPE(22:6/0:0) | 524.27794 | C_27_H_43_NO_7_P | [M-H]^-^ | 1.05 | 6.14E-03 |

* representative compared with the reference standards.

**Table S3**

The AUCs value of candidate liver injury biomarkers.

| **NO** | **Biomaker** | **AUC** | **P-value** |
| --- | --- | --- | --- |
| 1 | Kynurenine | 0.9844 | 1.10E-03 |
| 2 | Sphinganine | 0.9184 | 8.80E-03 |
| 3 | Phytosphingosine | 0.8594 | 1.57E-02 |
| 4 | LysoPC(14:0) | 0.9531 | 2.30E-03 |
| 5 | LysoPC(15:0) | 0.8214 | 3.72E-02 |
| 6 | LysoPE(0:0/18:0) | 0.9286 | 1.01E-02 |
| 7 | LysoPC(16:1(9Z)) | 0.9531 | 2.30E-03 |
| 8 | Indoleacrylic acid | 1.00 | 8.00E-04 |
| 9 | Pyruvate | 1.00 | 1.20E-03 |
| 10 | Lactic acid | 0.9464 | 3.80E-03 |
| 11 | p-Cresol sulfate | 0.8333 | 3.89E-02 |
| 12 | Glycocholic Acid | 0.9286 | 5.50E-03 |
| 13 | Cholesterol sulfate | 0.8594 | 1.57E-02 |
| 14 | Taurocholic acid | 0.9375 | 3.30E-03 |
| 15 | Taurochenodeoxycholic acid | 0.875 | 2.01E-02 |
| 16 | LysoPE(22:6/0:0) | 0.8393 | 2.79E-02 |


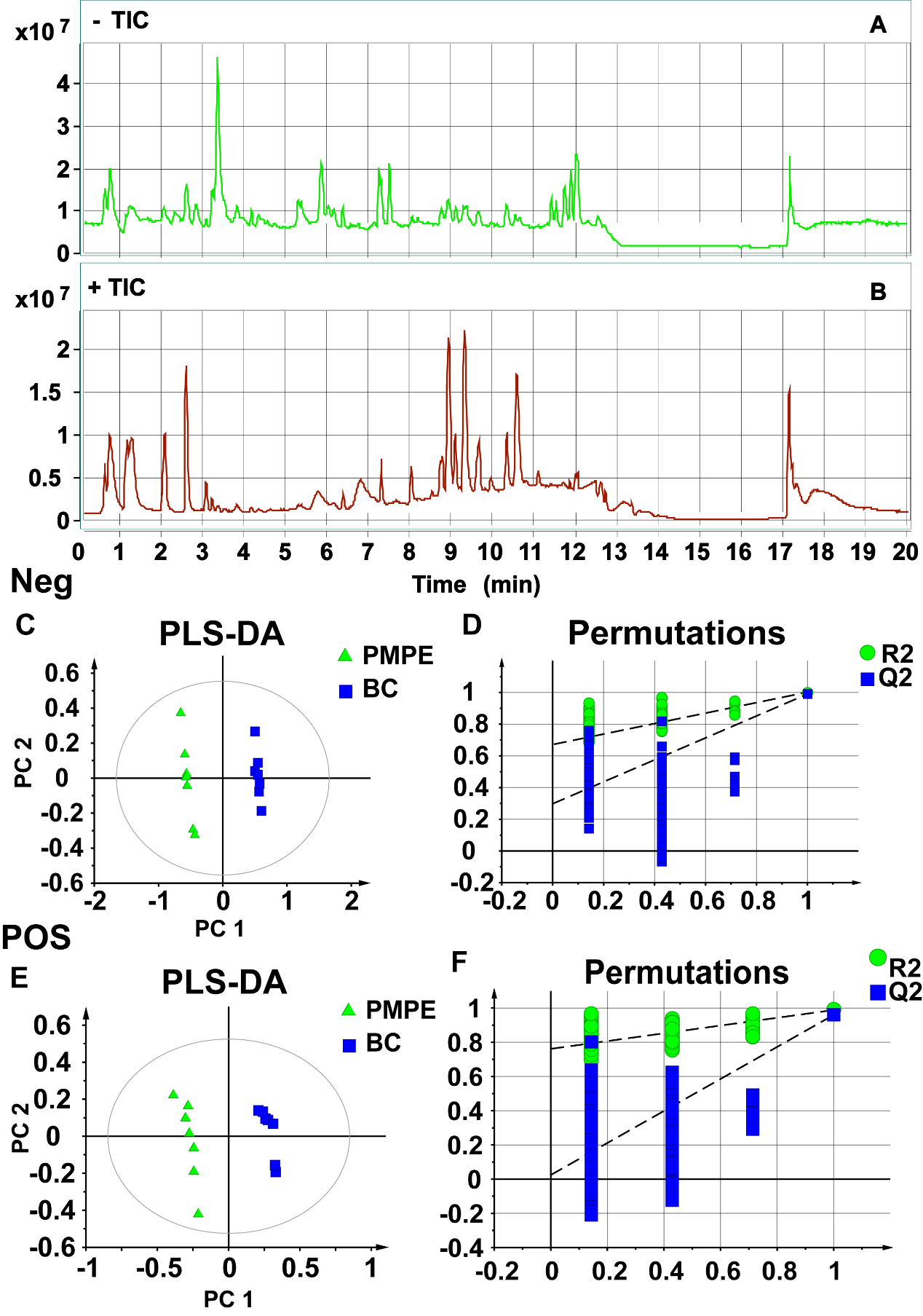


**Figure S1** TIC of the rat plasma from LC-MS spectra negative mode (A) and positive mode(B); PLS-DA analysis(C, E), permutations analysis(D, F) in negative mode and positive mode.


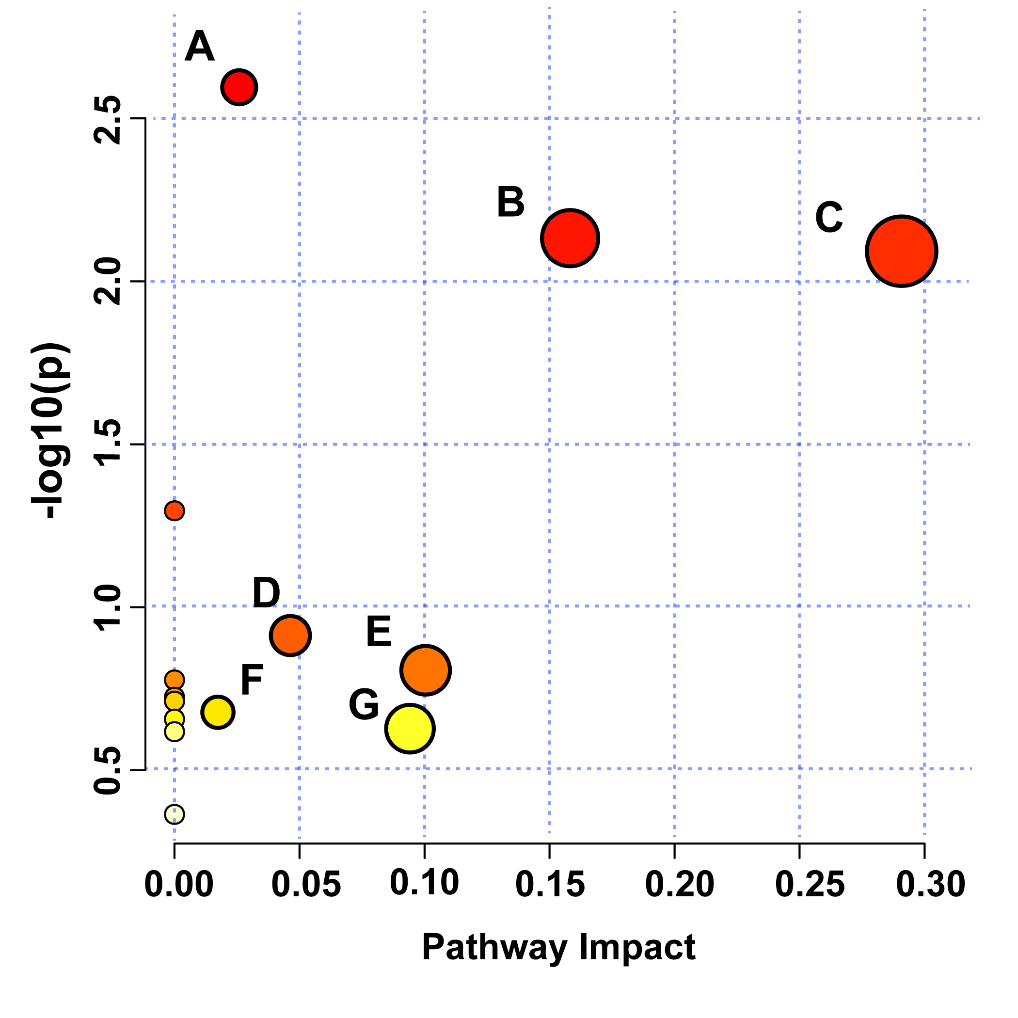


**Figure S2** Analysis of metabolic pathways related to liver injury. **A**: Primary bile acid biosynthesis, **B**: Sphingolipid metabolism, **C**: Pyruvate metabolism, **D**: Citrate cycle (TCA cycle), **E**: Glycolysis/Gluconeogenesis, **F**: Glycerophospholipid metabolism, **G**: Tryptophan metabolism.
